# Optimizing Arterial Tissue Thickness Measurement Protocols: Digital Vernier Caliper Versus Digital Thickness Gauge

**DOI:** 10.3390/mps7060090

**Published:** 2024-11-02

**Authors:** Alexandru Petru Ion, Alexandra Asztalos, Claudiu Constantin Ciucanu, Eliza Russu, Adrian Vasile Mureșan, Eliza-Mihaela Arbănași, Traian V. Chirilă, Gabriela Strnad, Emil-Marian Arbănași

**Affiliations:** 1 Doctoral School of Medicine and Pharmacy, George Emil Palade University of Medicine, Pharmacy, Science and Technology of Targu Mures, 540139 Targu Mures, Romania; peti.ion@outlook.com (A.P.I.); arbanasi.eliza@gmail.com (E.-M.A.); emilarbanasi1@gmail.com (E.-M.A.); 2 Regenerative Medicine Laboratory, Centre for Advanced Medical and Pharmaceutical Research (CCAMF), George Emil Palade University of Medicine, Pharmacy, Science and Technology of Targu Mures, 540139 Targu Mures, Romania; traian.chirila@qei.org.au; 3 Faculty of Medicine, George Emil Palade University of Medicine, Pharmacy, Science and Technology of Targu Mures, 540139 Targu Mures, Romania; aleztalos97@gmail.com; 4 Department of Vascular Surgery, George Emil Palade University of Medicine, Pharmacy, Science and Technology of Targu Mures, 540139 Targu Mures, Romania; eliza.russu@umfst.ro (E.R.); adrian.muresan@umfst.ro (A.V.M.); 5 Clinic of Vascular Surgery, Mures County Emergency Hospital, 540136 Targu Mures, Romania; 6 Queensland Eye Institute, Woolloongabba, South Brisbane, QLD 4102, Australia; 7 School of Chemistry and Physics, Queensland University of Technology, Brisbane, QLD 4001, Australia; 8 Australian Institute of Bioengineering and Nanotechnology (AIBN), University of Queensland, St. Lucia, QLD 4072, Australia; 9 Department of Industrial Engineering, Faculty of Engineering and Information Technology, George Emil Palade University of Medicine, Pharmacy, Science and Technology of Targu Mures, 540139 Targu Mures, Romania; gabriela.strnad@umfst.ro

**Keywords:** tissue thickness, vernier caliper, thickness gauge, protocol, arterial tissue

## Abstract

Background: The aim of this study is to analyze the reproducibility of sample thickness measurements taken by a non-experienced user by comparing a standard digital vernier caliper, with four different protocols, to a specialized thickness gauge. Methods: The current study is a methodological study where we examined the thickness of the porcine arterial wall in the thoracic aorta of six pigs. Two adjacent samples of 10 × 10 mm from each aorta were excised longitudinally from the anterior wall, resulting in twelve specimens. Five protocols were employed to measure the thickness of each sample. In four of these protocols, digital vernier calipers (Multicomp PRO MP012475) were utilized, while the fifth protocol utilized a specialized digital thickness gauge (Mitutoyo 547-500S, Mitutoyo Corp., Kawasaki, Japan). Results: We observed a higher average thickness of the samples during the initial measurement compared to the second measurement (1.11 ± 0.16 vs. 0.94 ± 0.17, *p* = 0.0319) with the first protocol and smaller values than those determined at the last measurement (0.93 ± 0.15 vs. 1.10 ± 0.15, *p* = 0.0135) for the third protocol. Further, with the digital vernier calipers, we recorded lower values for all four protocols than for the digital thickness gauge determinations. In addition, we computed the ratio of the thicknesses measured during the first, second, and third measurements to analyze how consistent the values were across the three consecutive measurements, with no difference regarding the third, fourth, and control protocols. Conclusions: The digital thickness gauge offers dependable measurements, regardless of the user’s expertise in assessing tissue thickness, and demonstrates a substantially higher reproducibility when compared to the digital vernier. We also found that taking an average of the thickness measurements from four specific points on each half of the sides or on each diagonal of each corner yielded consistently reliable results over time when using a standard digital vernier caliper instead of a specialized one.

## 1. Introduction

Cardiovascular diseases represent the first cause of mortality worldwide, although recently, there has been a trend of decreasing incidence and morbidity [1,2]. The most common cardiovascular diseases include coronary atherosclerosis [3], stroke [4], and peripheral arterial disease (PAD) [3,5]. While the decision for surgical or endovascular treatment in these conditions has traditionally been based on the severity of intra-arterial stenosis [6,7], a study by Mortensen et al. [8] revealed that plaque burden, rather than stenosis severity, is a more accurate predictor of cardiovascular events and mortality. Numerous studies have examined the appearance and development of atherosclerotic plaques due to local hemodynamic changes in arterial flow, shear stress in the arterial wall, and biomechanical characteristics [9,10,11,12,13,14,15].

Researchers have become interested in studying the biomechanical behavior of vascular tissue due to its significance in developing new stents and stent-grafts for conditions such as obstructive coronary disease, PAD, and abdominal aortic aneurysms (AAAs) [16,17,18]. However, the inhomogeneous wall thickness can affect the accuracy of biomechanical characteristics [19,20,21]. Various tools such as digital vernier calipers, micrometers, or thickness gauges [22,23] are utilized to determine the tissue thickness. However, the results may not be consistent, and the user’s lack of experience can impact the overall results [24,25]. Thus, new studies are required to analyze and propose new protocols for determining the thickness of the specimens using different instruments.

The aim of this study is to analyze the reproducibility and consistency of sample thickness measurements performed by a non-experienced user, using a standard digital vernier caliper with four different protocols and comparing this to a specialized thickness gauge. Additionally, we aim to determine which of the four methods demonstrates the highest reproducibility and which shows values similar to those generated by the specialized thickness gauge.

## 2. Materials and Methods

### 2.1. Porcine Thoracic Aortic Sample

The current study is a methodological study where we examined the thickness of the porcine arterial wall in the thoracic aorta of six pigs. The aortic segments were obtained from 20-week-old porcine specimens from a local slaughterhouse. All the animals were male and weighed 40–50 kg, with the aortic segment taken from the same anatomical position to minimize the risk of variability in aortic wall thickness. The tubular segment of the thoracic aorta was immediately transported to the laboratory for further processing. Two adjacent samples of 10 × 10 mm from each aorta were excised longitudinally from the anterior wall, resulting in twelve specimens. The specimens were stored in phosphate-buffered saline (PBS) for 1 h before measurements.

### 2.2. Measurement Technique

Five protocols were employed to measure the thickness of each sample. In four of these protocols, digital vernier calipers (Multicomp PRO MP012475) were utilized, while the fifth protocol utilized a specialized digital thickness gauge (Mitutoyo 547-500S, Mitutoyo Corp., Kawasaki, Japan). To minimize the risk of bias, each of the examinations and protocols were carried out by the same individual who had no prior experience with the two types of digital calipers. Moreover, after each protocol an assistant changed the order of the samples. This was carried out to ensure that the operator could measure the samples blindly when following the protocols, without being influenced by the previous determinations. Additionally, the thickness was expressed in millimeters (mm).

**Figure 1:**
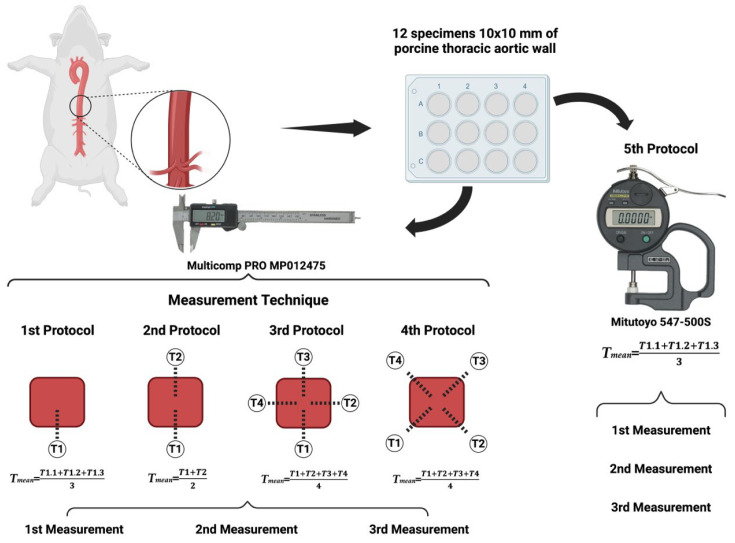
The graphic representation of the two types of digital calipers used in this study, as well as the five protocols for analyzing the thickness of porcine thoracic aorta specimens.

In the first protocol, we measured the thickness of the samples by taking three measurements of a single side of each specimen and then calculating the average of these measurements as the final thickness of the sample (Figure 1—First protocol). In the second protocol, we measured the thickness of two random opposite sides of the sample and then calculated the average as the final thickness (Figure 1—Second protocol). In the third protocol, we measured the thickness at the midpoint of all sides of the specimens and then calculated the average as the overall thickness of the sample (Figure 1—Third protocol). In the fourth protocol, we determined the thickness of each corner and then calculated the average of the four determinations as the final thickness of the sample (Figure 1—Fourth protocol). Finally, in the fifth protocol, we used the Mitutoyo 547-500S digital thickness gauge to determine the sample thickness as the average of three consecutive measurements (Figure 1—Fifth protocol). Each protocol was performed three times, in order to analyze its reproducibility.

Moreover, we used a reference protocol to determine the samples’ thickness using an optical microscope (Optika B—383 MET (Optika, Ponteranica, Italy)) equipped with a Nikon D3400 digital camera (Nikon, Ayutthaya, Thailand). To avoid variation in porcine aortic wall thickness, we used 10 × 10 mm 3D printed square samples.

### 2.3. Three-Dimensional (3D) Printing Samples

The 10 × 10 mm samples were 3D printed on the Ultimaker S3 3D printer (Ultimaker, Zaltbommel, Netherlands) from TPU 85A material using a filament with a thickness of 2.85 mm. The preset thickness of the samples was 8 mm. These samples were used to determine which of the five protocols proposed in the current study presented a similar accuracy to the optical microscopy protocol.

### 2.4. Study Outcome

The main endpoint was to analyze the reproducibility and accuracy of the samples’ thickness by comparing the measurements taken with a standard digital vernier caliper and a specialized digital thickness gauge.

### 2.5. Statistical Analysis

In this study, we performed a statistical analysis using SPSS for MacOS version 29.0.1.1 (Chicago, IL, USA). The average thickness of the specimens were reported as mean values and standard deviations. We used the One-way ANOVA with the Dunnett multiple comparisons test to compare the differences in thickness values between protocols. In addition, we used the same test to analyze the differences in ratios for the first four protocols compared to the ratio of the control group. To quantitatively evaluate the differences in variability among the five measurement protocols, we conducted Levene’s test for equality of variances. This statistical test was employed to compare the variability in thickness measurements between the initial four protocols and the control protocol, since variability is a crucial factor in establishing the reliability of thickness measurements. We considered a *p* value of less than 0.05 to be statistically significant.

## 3. Results

**Figure 2:**
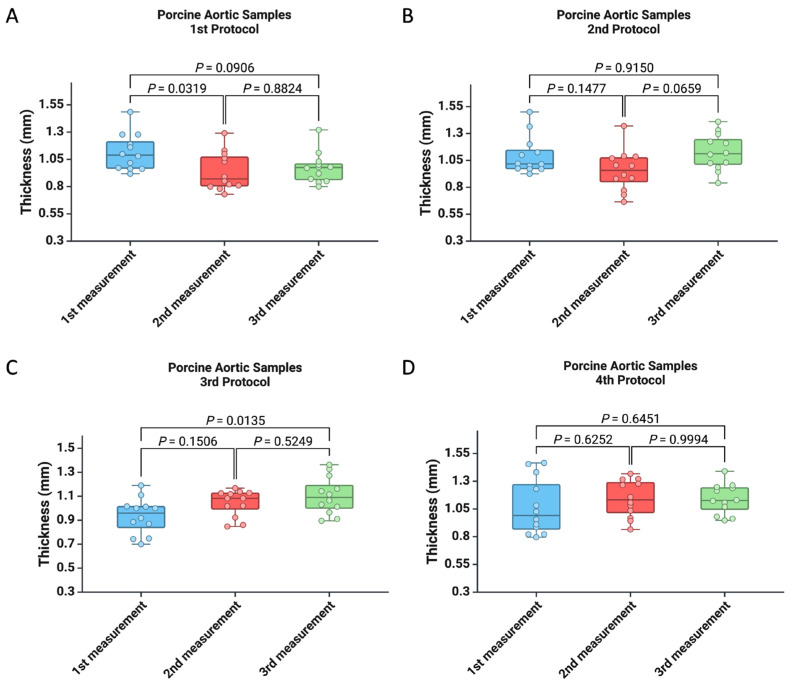
Comparison of the thickness of the specimens between the three consecutive measurements using the standard digital vernier caliper with (**A**) the first protocol, (**B**) the second protocol, (**C**) the third protocol, and (**D**) the fourth protocol.

In the first part of this study, we compared the thickness differences of the specimens obtained from three consecutive analyses (Figure 2). According to the first protocol, we observed a higher average thickness of the samples during the initial measurement compared to the second measurement (1.11 ± 0.16 vs. 0.94 ± 0.17, *p* = 0.0319) (Figure 2A). For the third protocol, we found that the thickness of the samples determined at the first measurement were smaller than those determined at the last measurement (0.93 ± 0.15 vs. 1.10 ± 0.15, *p* = 0.0135) (Figure 2C). No significant differences were observed in the measurements for the second and fourth protocols (Figure 2B,D).

**Figure 3:**
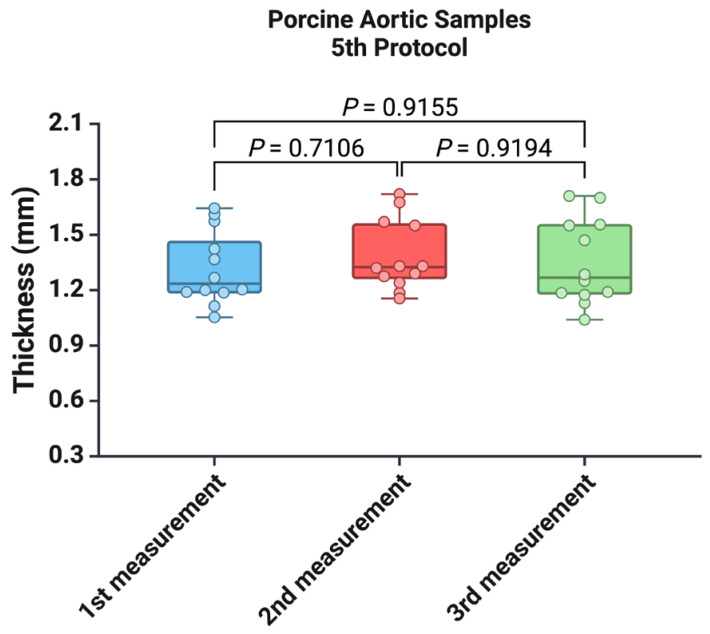
Comparison of the thickness of the specimens between the three consecutive measurements using the specialized digital thickness gauge.

Additionally, we measured the thickness of the samples with the Mitutoyo 547-500S digital thickness gauge through three consecutive determinations, during which we recorded similar values, as seen in Figure 3.

In addition, we compared the five protocols to the optical microscopy reference protocol to determine which one had the best accuracy. To avoid the inhomogeneous wall thickness of the animal aorta samples, we decided to 3D print ten samples measuring 10 × 10 mm with a preset thickness of 8 mm. We used the five proposed protocols and optical microscopy to measure the thickness of the samples. Our findings showed that the first four protocols had significantly higher thickness values when compared to optical microscopy (*p* < 0.05 for all), while there were no differences when compared to the fifth protocol (*p* = 0.4226) (Supplementary Figure S1).

**Figure 4:**
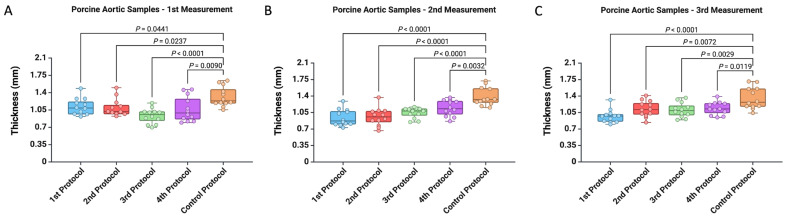
Comparison of the five protocols for measuring arterial wall thickness for (**A**) the first measurement, (**B**) the second measurement, and (**C**) the third measurement.

Based on the reproducibility of the three measurements using a specialized thickness gauge, we used these values as a control to determine which of the four protocols using a standard digital vernier caliper provides the best accuracy. Figure 4 shows that we recorded lower values for the thickness of porcine aortic samples for all four protocols regardless of the determination time (all *p* < 0.05). However, in the first measurement, we observed the smallest difference between the first protocol and the control protocol (*p* = 0.044) (Figure 4A). For the second and third measurements of the samples, the smallest difference was between the fourth protocol and the control (*p* = 0.0032 and *p* = 0.0119) (Figure 4B,C).

**Figure 5:**
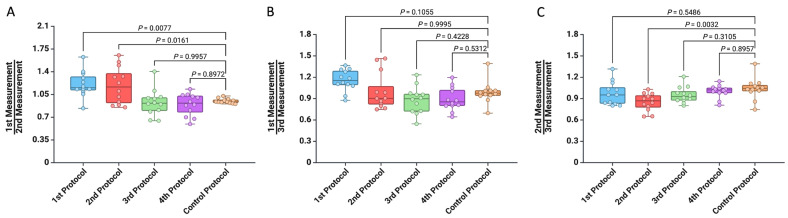
Comparison of the five protocols for measuring arterial wall thickness for (**A**) the ratio between the first and second measurements, (**B**) the ratio between the first and third measurements, and (**C**) the ratio between the second and third measurements.

In addition, we computed the ratio of the thicknesses measured during the first, second, and third measurements to analyze how consistent the values were across the three consecutive measurements. As shown in Figure 5, the thickness measurements from the third and fourth protocols did not differ from the control protocol for all three ratios.

Furthermore, we conducted Levene’s test to evaluate the differences in variability between the first four protocols and the control protocol. The results indicated a significant difference (*p* < 0.05 across all comparisons) in variability among the protocols for the ratio between the first and second measurements, suggesting the presence of notable differences in variability that warrant consideration. However, no significant differences were reported for the ratios between the first and third measurements or between the second and third measurements (Table 1).

## 4. Discussion

The main result of this study is the demonstration, for the first time to our knowledge, that the Mitutoyo 547-500S digital thickness gauge shows a significantly greater reproducibility than the digital vernier gauges, which is independent of the user’s experience in measuring tissue thickness. Our research also indicated that taking an average of the thickness measurements from four specific points on each half of the sides (Third protocol) or on each diagonal of each corner (Fourth protocol) consistently produced reliable results over time when using the standard digital vernier caliper as opposed to the specialized one.

The biomechanical analysis of vascular tissue can provide valuable information about how it behaves under different conditions, from normal stretches to excessive stretching and failure [26,27,28,29]. By analyzing the arterial wall and the specific biomechanical profile of each segment along the entire length of an artery, researchers can develop new, more effective stents with improved long-term permeability [29]. However, the specimens’ thickness, width, and size can influence the biomechanical characteristics and thus generate erroneous data [30,31,32,33]. Raghavan et al. [30] analyzed the regional distribution of wall thicknesses in AAA and found values ranging from 0.23 mm to 4.26 mm, with lower values for the posterior and right lateral walls. Marais et al. [31] showed that thickness and diameter indicate a higher risk of rupture in an AAA xenograft animal model. Moreover, human vascular tissue specimens vary in thickness and structure based on their age, the anatomical segments, and risk factors [30,31,32,33]. The correct determination of tissue thickness was also demonstrated by de Gelidi et al. [23], showing its important impact on numerical arterial models.

The current study has some important limitations. Firstly, the small number of porcine thoracic aortic wall samples analyzed means that any generalizations based on our data should be made with caution. Secondly, the same person performed all five protocols without prior experience of using the two types of digital calipers, which may have led to an increased precision of the values recorded with the vernier caliper due to an improved user performance. Thirdly, a significant limitation of this study is the lack of a gold-standard method for determining the thickness of porcine aortic specimens, which is necessary to ascertain the accuracy of the two instruments analyzed, and more specifically the five proposed protocols. Finally, another limitation is the lack of biomechanical analysis needed to understand how different thickness determination methods might affect the biomechanical profile. Therefore, we suggest that further studies should explore the role of the user’s experience with different instruments for measuring the thickness of vascular specimens and the impact of these determinations on the biomechanical characteristics of the samples.

## 5. Conclusions

The digital thickness gauge offers dependable measurements, regardless of the user’s expertise in assessing tissue thickness, and demonstrates a substantially higher reproducibility compared to the digital vernier. We discovered that averaging the thickness measurements from four specific points on each side or along each diagonal of the corners tended to give us consistent and reliable results over time, especially when we used a standard digital vernier caliper instead of a specialized one. Additionally, this study emphasized that a routine procedure, such as measuring tissue thickness, may yield inconsistent results if the user lacks experience, potentially causing significant errors in assessing biomechanical properties.

## Figures and Tables

**Table 1:** Analysis of variability differences between the first four protocols and the control protocol.

2nd/3rd Measurement		1st/3rd Measurement		1st/2nd Measurement		Ratios
Levene’s Test *p*-value (Comparison with Control)	Thickness mean ± SD (mm)	Levene’s Test *p*-value (Comparison with Control)	Thickness mean ± SD (mm)	Levene’s Test *p*-value (Comparison with Control)	Thickness mean ± SD (mm)	Protocols
0.385	0.97 ± 0.16	0.510	1.16 ± 0.15	0.003	1.21 ± 0.21	1st Protocol
0.885	0.85 ± 0.12	0.057	1.01 ± 0.26	<0.001	1.18 ± 0.28	2nd Protocol
0.852	0.95 ± 0.12	0.262	0.88 ± 0.19	0.010	0.93 ± 0.20	3rd Protocol
0.444	1.01 ± 0.09	0.376	0.89 ± 0.16	<0.001	0.89 ± 0.17	4th Protocol
-	1.03 ± 0.15	-	0.98 ± 0.15	-	0.95 ± 0.04	Control Protocol

## Data Availability

The data that support the findings of this study are available from the corresponding author upon reasonable request.

## Supplementary Materials

The following supporting information can be downloaded at: https://www.mdpi.com/article/10.3390/mps7060090/s1, Figure S1: Comparison of thickness measurements of 3D-printed aorta wall samples using five protocols versus Optical microscopy reference.
