# Supplementary material for: Optimizing Arterial Tissue Thickness Measurement Protocols: Digital Vernier Caliper Versus Digital Thickness Gauge
Source: Methods Protoc. 2024 Nov 2;7(6):90. doi: 10.3390/mps7060090 (PMC11587071; doi:10.3390/mps7060090)
Supplement: Supplementary file 1 [file mps-07-00090-s001.zip › mps-3146384-supplementary.pdf]

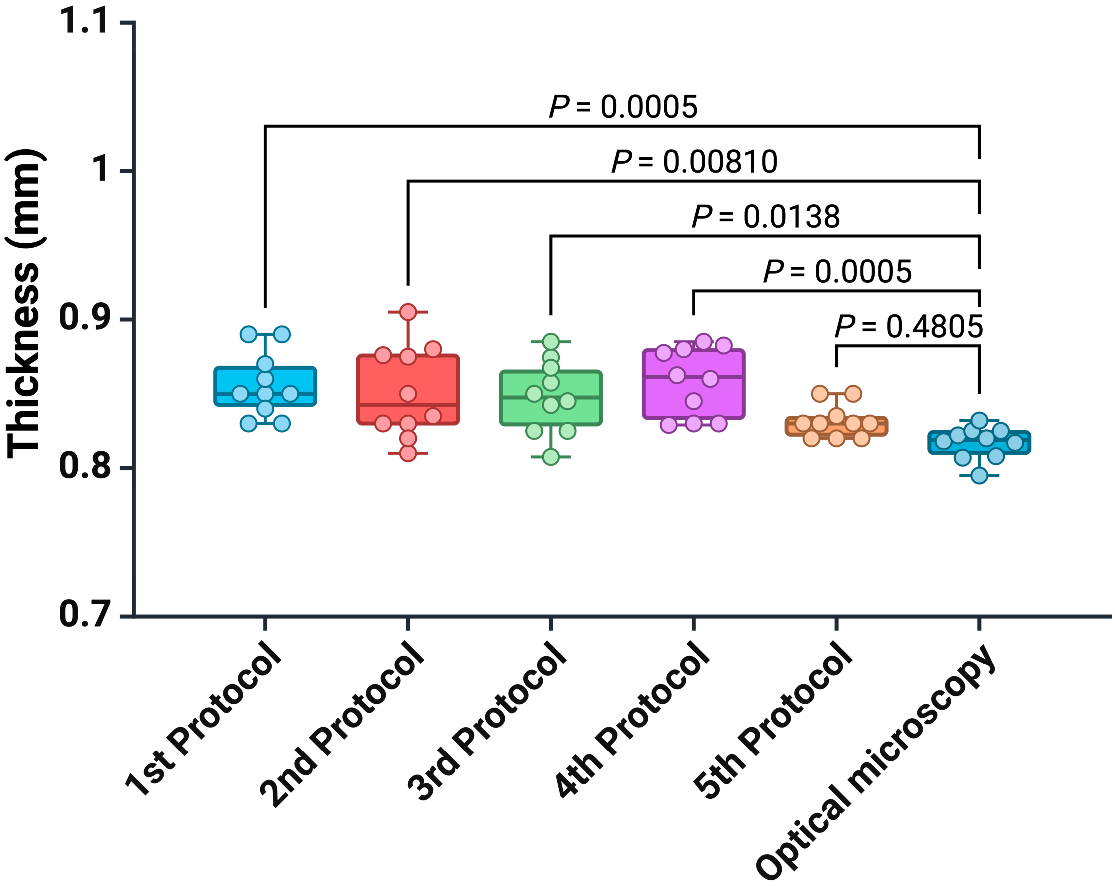

Figure S1. Comparison of thickness measurements of 3D-printed aorta wall samples using five protocols versus Optical microscopy reference.
